# Use of the Highly Biocompatible Au Nanocages@PEG Nanoparticles as a New Contrast Agent for In Vivo Computed Tomography Scan Imaging

**DOI:** 10.1186/s11671-020-3286-2

**Published:** 2020-03-04

**Authors:** Yan Gao, Jian Kang, Zhen Lei, Yankun Li, Xifan Mei, Guannan Wang

**Affiliations:** 1 The First Affiliated Hospital of Jinzhou Medical University, Jinzhou, 121001 China; 2 College of Pharmacy, Jinzhou Medical University, Jinzhou, 121001 China; 3 The Key Laboratory for Medical Tissue Engineering, College of Medical Engineering, Jining Medical University, Jining, 272067 China

**Keywords:** Biocompatibility, AuNC@PEGs, Contrast agent, In vivo CT imaging

## Abstract

In recent years, contrast agents have been widely used in imaging technology to improve quality. Nanoparticles have better in vivo detection capability than conventional molecular scale contrast agents. In this study, a new type of Au nanocages@PEG nanoparticles (AuNC@PEGs) with a strong X-ray absorption coefficient was synthesized as a contrast agent for computed tomography (CT) scan imaging. Results showed that AuNC@PEGs had good aqueous dispensation, low cytotoxicity, and strong X-ray absorption ability. Furthermore, in vivo studies have shown that the synthesized AuNC@PEGs have an evident contrast enhancement, long circulation time in the blood, and negligible toxicity in vivo. Therefore, the synthesized functionalized AuNC@PEGs in this study have great potential for clinical application in CT scan imaging.

## Introduction

In recent years, computed tomography (CT) scan has been the most commonly used diagnostic imaging technique in clinical settings, and it has a wide application in the study of various human tissues. Due to the strong penetrating and high contrast ability and relatively simple image processing of CT scan, it is considered the most powerful noninvasive imaging diagnostic technique in the modern medical system [1, 2]. However, in the process of imaging, there is no natural contrast between the lesion and some surrounding structures. Thus, a contrast agent, which is a substance with a relatively higher or lower density, must be used to distinguish the target structure or tissue and organs. Moreover, this substance has varying absorption capabilities in different tissues and can be observed via X-ray irradiation in the soft tissues. The use of some molecules and several microparticle contrast agents in CT scan imaging has been assessed [3–5].

At present, the most commonly used contrast agent for CT scan is an organic molecule containing iodine. Iodized molecules, such as iodide ion or non-ionic preparation, are widely used as a contrast agent for CT scans in clinical settings. Although the iodized molecules can provide good CT scan contrast enhancement, they have a fast renal clearance rate, a short circulation time in the body, and allergenic properties, which significantly limit further applications [6, 7]. Due to the rapid removal of the iodine developer, the effective time window of blood pool imaging is seriously limited, and a high-contrast image is difficult to obtain. Furthermore, the rapid clearance of a large dose of drugs may have potential side effects in the kidney [8, 9].

In the last decade, the application of nanoparticles in biomedicine, particularly in diagnostic imaging, has received considerable attention [10]. Compared with iodine-based contrast agents, nanoparticles have payloads of contrast characteristics that small molecules do not possess, and they also have a specific size, shape, and surface [11, 12]. Generally, nanoparticles with a large atomic number, such as gold, silver, and other metal nanoparticles, have an excellent X-ray absorbing coefficient; thus, they have a remarkable contrast enhancement capability [13, 14]. Among these nanoparticles, gold nanoparticles have been rapidly developed in the field of biomedicine and considered a substitute for iodine-based imaging agents due to their significant biological inertia and ease of synthesis and surface modification [15–17]. Gold nanoparticles have longer blood circulation time, lower risk of nephrotoxicity, and stronger X-ray absorption coefficient than iodine compounds. Therefore, a decreased dosage can be provided, and the risk of side effects is low [18]. Several gold nanoparticles, including nanospheres, nanorods, and nanostars, have been widely used as a contrast agent for CT scan imaging [19, 20], and it has a promising effect. Among the various gold nanostructures, the Au nanocages have a hollow interior and thin porous wall; thus, they have a higher surface area and more effective CT scan imaging ability than other gold nanoparticles with different morphologies [21, 22]. In recent years, Au nanocages have been used as a contrast enhancer for optical coherence tomography and photoacoustic tomography and have been found to have good performance. Meanwhile, due to the large absorption area of the Au nanocages, they are also effective photothermal transducers [23, 24]. To the best of our knowledge, only a few studies have assessed the use of Au nanocages as a contrast agent for CT scan imaging. Based on the abovementioned studies, we further explored the use of Au nanocages as a CT scan contrast agent. The application of nanoparticles in CT scan imaging requires surfaces with biocompatibility and biological activities. Polyethylene glycol (PEG) is a biodegradable and biocompatible polymer, which is also the stealth material used to prevent capture by RES and to improve biocompatibility, kidney scavenging ability, and blood circulation time; thus, PEGylated nanoparticles can be retained in the blood for a long period [15, 25–28].

In this study, new AuNC@PEGs were prepared and characterized. Then, the in vitro biocompatibility of AuNC@PEGs was evaluated by MTT colorimetry, lactate dehydrogenase (LDH) leakage method, intracellular reactive oxygen species (ROS) concentration assay, Calcein-AM/PI, and other experimental techniques. In addition, hematological and histological analyses were performed to determine the toxicity of AuNC@PEGs in vivo. Results showed that AuNC@PEGs had great in vitro and in vivo biocompatibility. Moreover, AuNC@PEGs were found to have a stronger in vitro and in vivo CT scan imaging ability. These experimental results showed that the synthesized AuNC@PEGs have obvious advantages, such as strong contrast, long blood circulation time, and low risk of nephrotoxicity. Therefore, the synthesized functionalized AuNC@PEGs in this study have great potential for clinical application in CT scan imaging.

## Methods

All experimental protocols, including any relevant details, were approved by the Regional Ethics Committee of Jinzhou Medical University in Liaoning Province, China.

### Materials and Instruments

The LDH and ROS test kits were purchased from the Nanjing Institute of Bioengineering, China, and the Calcein-AM/PI staining kits from Shanghai Dongren Chemical Technology Co., Ltd., China. Other chemical agents and solvents were purchased from Sigma-Aldrich. All sections were assessed using a fluorescence microscope (DMI4000B, Leica, Wetzlar, Germany). The characteristics of the synthesized nanoparticles were evaluated with a transmission electron microscope (TEM). A 256-row, 512-slice spiral CT scan (Philips, Germany) was utilized, and the imaging parameters were as follows: slice thickness, 0.625 mm; tube voltage, 100 Kvp; and tube current, 100 mA.

### Synthesis of AuNC@PEGs

Au nanocages were prepared using a simple galvanic replacement reaction between Ag nanocubes and HAuCl_4_ solution, according to a previous study [29, 30]. Typically, 25-nm Ag nanocubes were prepared in diethylene glycol and were used as templates for the synthesis of 30-nm Au nanocages. Then, SH-PEG (MW ≈ 2000, 10 mg dissolved in 5 mL of phosphate-buffered saline (PBS)) was added to the AuNC solution (pH 8.0, 6.55 nM, 6 mL) and was stirred overnight in the dark under nitrogen protection. After washing the AuNC@PEGs for three more times, they were dispersed in aqueous solution.

### Evaluation of In Vitro Toxicity

In this study, MTT colorimetry, LDH leakage method, intracellular ROS concentration assay, and Calcein-AM/PI staining were used to detect the toxicity of the synthesized AuNC@PEGs in vitro. The HUVEC cells were inoculated into 96-well plates with a density of 1 × 10^4^/well. The RPMI-1640 medium supplemented with 10% fetal bovine serum and penicillin (100 μg/mL) and streptomycin was used. Then, the cells were cultured at 37 °C and 5% carbon dioxide incubator for 12 h. Then, the medium with AuNC@PEGs at different concentrations (10, 20, 50, 100, 200, 500, and 1000 μg/mL) was added for further culture. After 24 h, the MTT assay was obtained. The culture medium without nanoparticles was used as the control in each group.

Then, the content of lactate dehydrogenase (LDH) released from HUVEC cells treated with AuNC@PEGs at different concentrations was determined to evaluate toxicity in vitro. The cells were inoculated similar to MTT and were then treated with AuNC@PEGs at different concentrations (10, 20, 50, 100, 200, 500, and 1000 μg/mL) for 24 h. Then, the supernatant was separated, centrifuged, and transferred to a clean 96-well plate. The activity of LDH in the culture medium was assessed according to the manufacturer’s instructions, and the absorbance was determined using an enzyme-labeling instrument (450 nm).

Based on the principle of measuring intracellular ROS concentration using the ROS kit, DCFH was oxidized to dichlorofluorescein (DCF), which is a strong green fluorescent substance DCF, in the presence of 2',7'-dichlorofluorescein (DCFH-DA). The HUVEC cells were cultured in 24-well plates for 12 h, treated with AuNC@PEGs at different concentrations (50, 100, 200, and 500 μg/mL) for 24 h, and incubated with DCFH-DA at 37 °C for 40 min. The cells treated with hydrogen peroxide (H_2_O_2_) were used as positive control. The fluorescence intensity of the cells was observed using a fluorescence microscope (λex, 485 nm; λem, 525 nm). Before assessment, a serum- and ice-free medium was used three times for washing.

For live/dead staining, the HUVEC cells were inoculated into 24-well plates and were cultured for 12 h. Then, the cells were treated with AuNC@PEGs at different concentrations (10, 20, 50, 100, 200, 500, and 1000 μg/mL) for 24 h. After digestion with trypsin-EDTA via centrifugation, the cells were washed with PBS (pH = 7.4); then, the prepared cell suspension was mixed with the pre-configured Calcein-AM/PI reagent and was cultured at 37 °C for 15 min. To detect the toxicity of AuNC@PEGs, the number of dead cells was assessed via fluorescence microscopy.

### Animal Model

All animal experiment procedures were performed according to the criteria established by the Animal Protection and Use Committee of Jinzhou Medical University. After the experiment, the animals were euthanized according to humanitarian principles. In this study, adult Sprague Dawley rats weighing 250–300 g (purchased from the Animal Center of Jinzhou Medical University) were used. In this experiment, all animals were randomly divided into groups. Chloral hydrate solution (10 wt%) was administered via the abdominal cavity; then, all materials were injected via the tail vein.

### In Vitro and In Vivo CT Scan Imaging

For in vitro CT scan imaging, AuNC@PEGs at different concentrations and iodine solutions were placed in EP tubes and were arranged in the proper order, and a CT scan was performed from front to back. In the in vivo CT scan, after administering anesthesia, the animals were scanned from head to tail, with the center of the abdominal cavity used as the landmark. The position of the animals did not change every time. All original data (0.625 mm image) were transmitted to the Philips workstation for analysis via CT scan.

### Evaluation of In Vivo Toxicity

Hematologic analysis was conducted using the standard saphenous vein blood collection technique. The tissues of the heart, liver, spleen, lung, and kidney of the rats were fixed with 4% paraformaldehyde for 48 h and were embedded in paraffin after dehydration. The paraffin section was 5 μm thick and was mounted on a glass slide. Hematoxylin and eosin (H&E) staining was then performed, and analysis was conducted under a microscope.

### Statistical Analysis

Data were analyzed using one-way analysis of variance, and *P* value was used as the index. A *P* value < 0.05 was considered statistically significant, as expressed by the average value of SD.

## Results and Discussion

### Synthesis and Characterization of the AuNC@PEGs

**Fig. 1:**
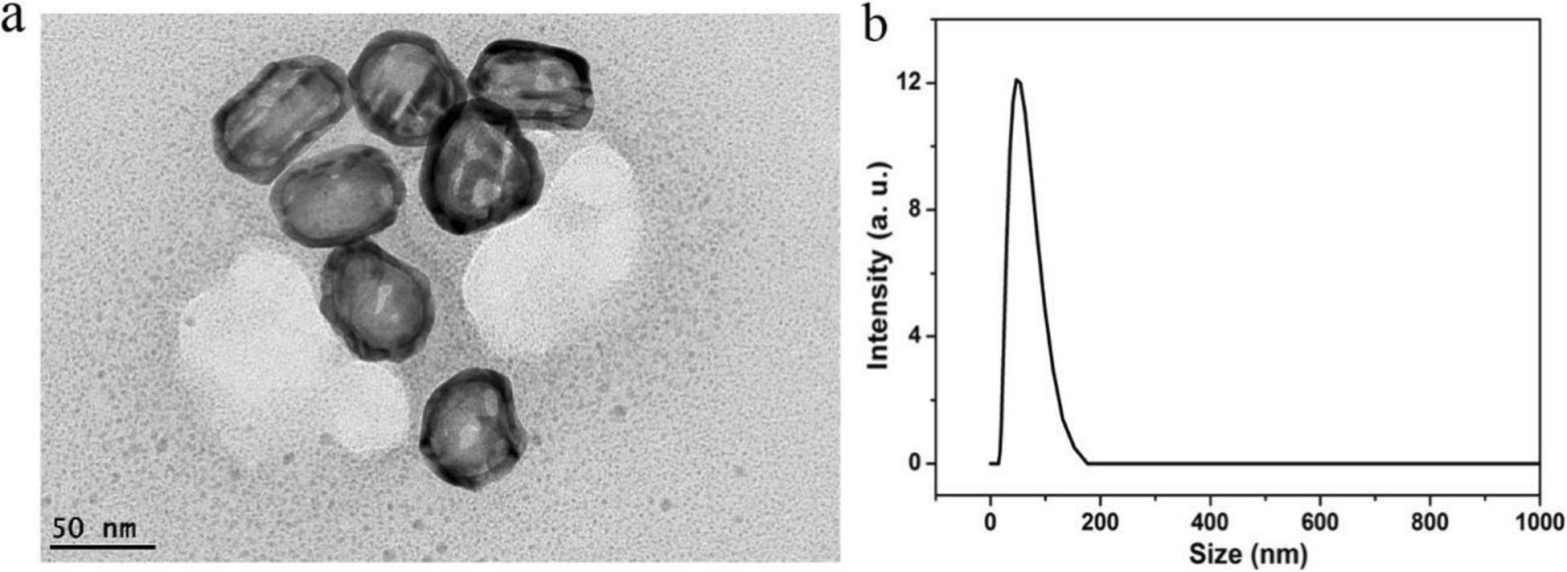
TEM images of AuNC@PEGs (**a**) and DLS of AuNC@PEGs (**b**)

Surface functionalization and size control are two key factors for the development of high-performance nano-contrast agent **[**15]. The structure and characters of AuNC@PEGs were determined by TEM and DLS. Fig. 1a showed the results that the size of AuNC@PEGs was around 40 nm with high uniformity; meanwhile, the hydration radius of AuNC@PEGs was also used to test the dispersion in the solution, as shown in Fig. 1b, the hydration radius of AuNC@PEGs was about 50 nm, showing that AuNC@PEGs were very stable without any aggregation. AuNC@PEGs have a smaller size and relatively good biological inertia, which are better for nanomedicine applications. Moreover, the hollow cage structure indicates large internal- and external-specific surface areas and better CT scan imaging ability, and the surrounding metal walls provided additional protection for payloads during its processing, transportation, and storage. With its obvious core-shell structure, the outside was covered with PEG of biological applicability, which can effectively enhance biocompatibility and escape macrophage capture.

### Safety and Stability of AuNC@PEGs In Vitro

**Fig. 2:**
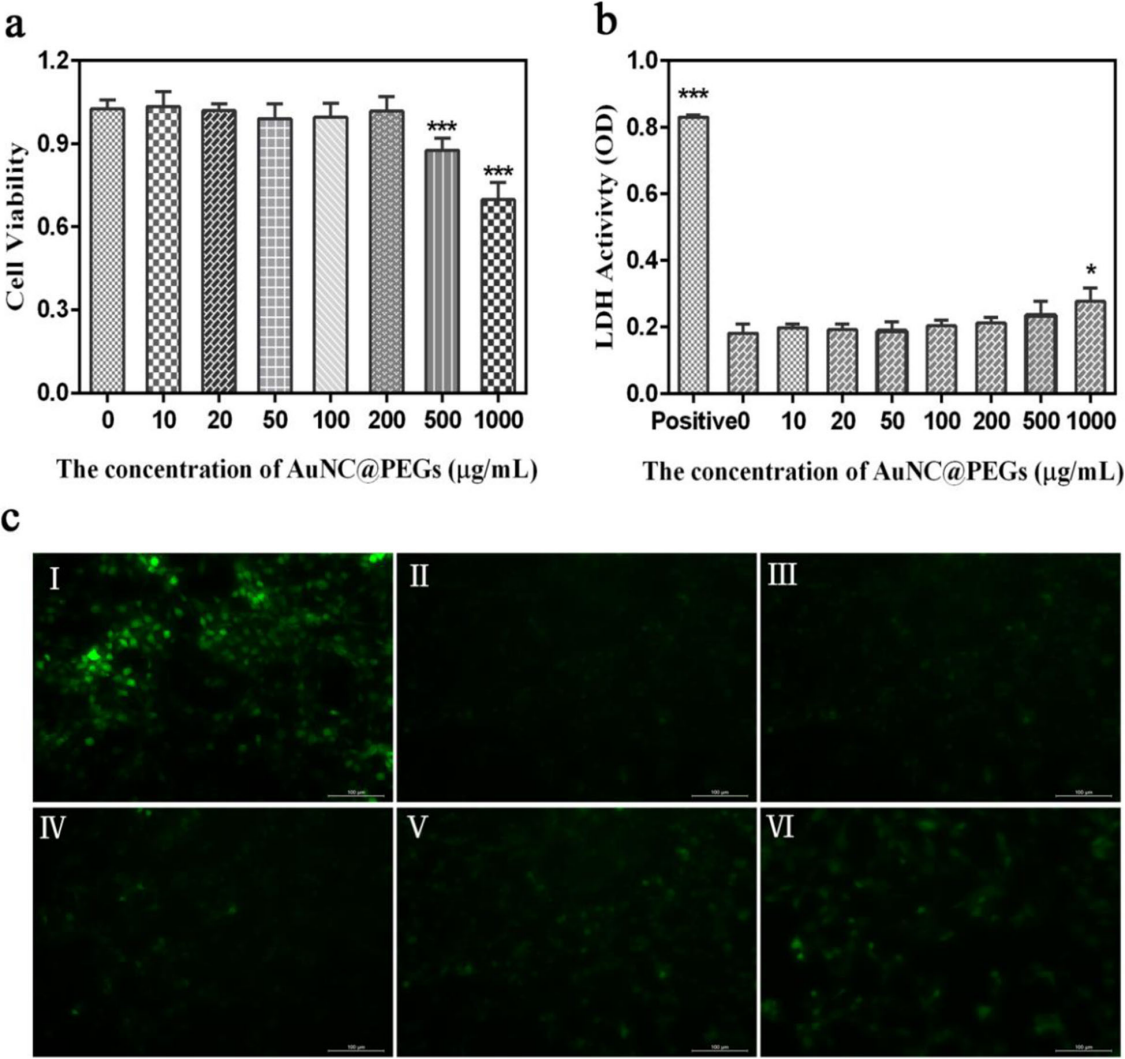
MTT evaluation of the viability of HUVEC cells cultured with different AuNC@PEGs concentrations for 24 h (**a**). Assessment of lactate dehydrogenase in supernatant induced by AuNC@PEGs with LDH (**b**). Examination of 24 h fluorescence imaging of cells cultured with AuNC@PEGs at different concentrations (H_2_O_2_ (I), 0 μg/mL (II), 50 μg/mL (III), 100 μg/mL (IV), 200 μg/mL (V) and 500 μg/mL (VI)) by ROS method (**c**). *P < 0.05, ***P < 0.001. Scale bars are 100 μm

Before using AuNC@PEGs for in vivo imaging, we evaluated their safety and stability. The effect of AuNC@PEGs on the viability of HUVEC cells was detected using the MTT assay (Fig. 2a). The cells were treated with AuNC@PEGs at different concentrations (10, 20, 50, 100, 200, 500, and 1000 μg/mL) for 24 h. The results of the MTT assay showed that the cell survival rate of the AuNC@PEGs was similar to that of the control group and it had favorable biocompatibility when the concentration of AuNC@PEGs reached 200 μg/mL. The cell survival rate at a concentration of 1000 μg/mL was still > 75%.

Furthermore, the LDH assay was also used to evaluate the biocompatibility of AuNC@PEGs in vitro. In normal cells, LDH is not allowed to pass through the cell membrane. When the cell membrane is damaged, LDH is released through the cell membrane **[**31]. Thus, we assessed the safety of AuNC@PEGs by measuring the LDH content in the cell supernatant (Fig. 2b) by treating cells with AuNC@PEGs at different concentrations for 24 h. Results showed that the LDH level released by the cells slightly increased compared to the unexposed control cells when the concentration of the AuNC@PEGs was < 200 μg/mL, and it was significantly lower than that of the positive control group (H_2_O_2_), which was consistent with the results of the MTT assay, and 200 μg/mL of AuNC@PEGs as optimal concentration were found to have good cytocompatibility.

**Fig. 3:**
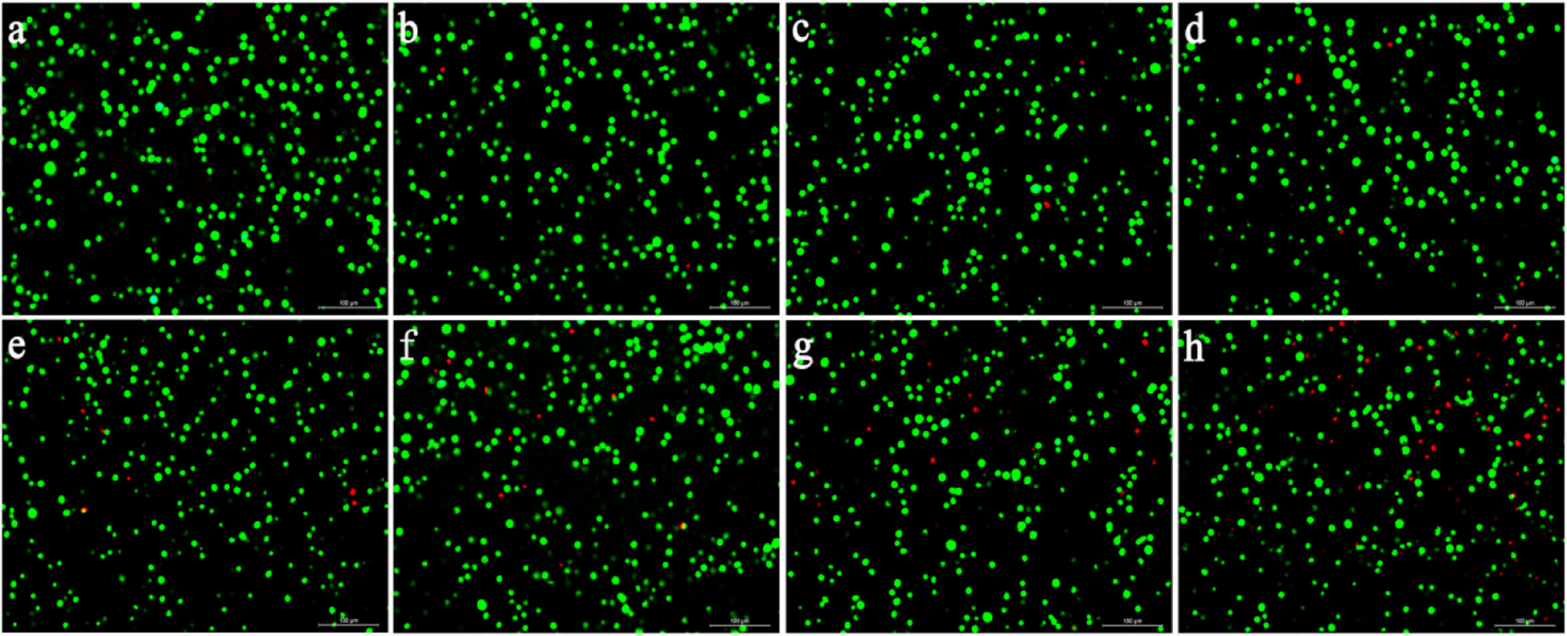
Fluorescence microscopy images of live and dead staining. Survival rate of HUVEC cells treated with AuNC@PEGs at different concentrations (0 μg/mL (**a**), 10 μg/mL (**b**), 20 μg/mL (**c**), 50 μg/mL (**d**), 100 μg/mL (**e**), 200 μg/mL (**f**), 500 μg/mL (**g**), and 1000 μg/mL (**h**)) for 24 h. Green fluorescence represents living cells and red fluorescence represents dead cells. The scale bars are 100 μm

Moreover, oxidative stress tests and assessment of live/dead cells immunofluorescence staining (Calcein-AM/PI) were performed to detect the toxicity of AuNC@PEGs in vitro. Oxidative stress is a harmful condition for all life systems, and excessive reactive oxygen species (ROS) can cause oxidative stress [32, 33]. Therefore, we measured the ROS level in the cells. After 24 h of induction by AuNC@PEGs at different concentrations, the green fluorescence intensity of the cells induced at a concentration of 50–200 μg/mL did not significantly differ from that of the control group, and it was significantly lower than that of the positive control group (Fig. 2c). The fluorescence intensity was proportional to the level of ROS. As shown in Fig. 3, the survival rate of HUVEC cells at a concentration of 0–200 μg/mL was > 90% (Fig. 3a–f). The abovementioned results validated that AuNC@PEGs at a concentration of 200 μg/mL are stable and have good cell compatibility, and they can be promising clinical contrast agents.

### In Vitro CT Scan Imaging and Determination of CT Value

**Fig. 4:**
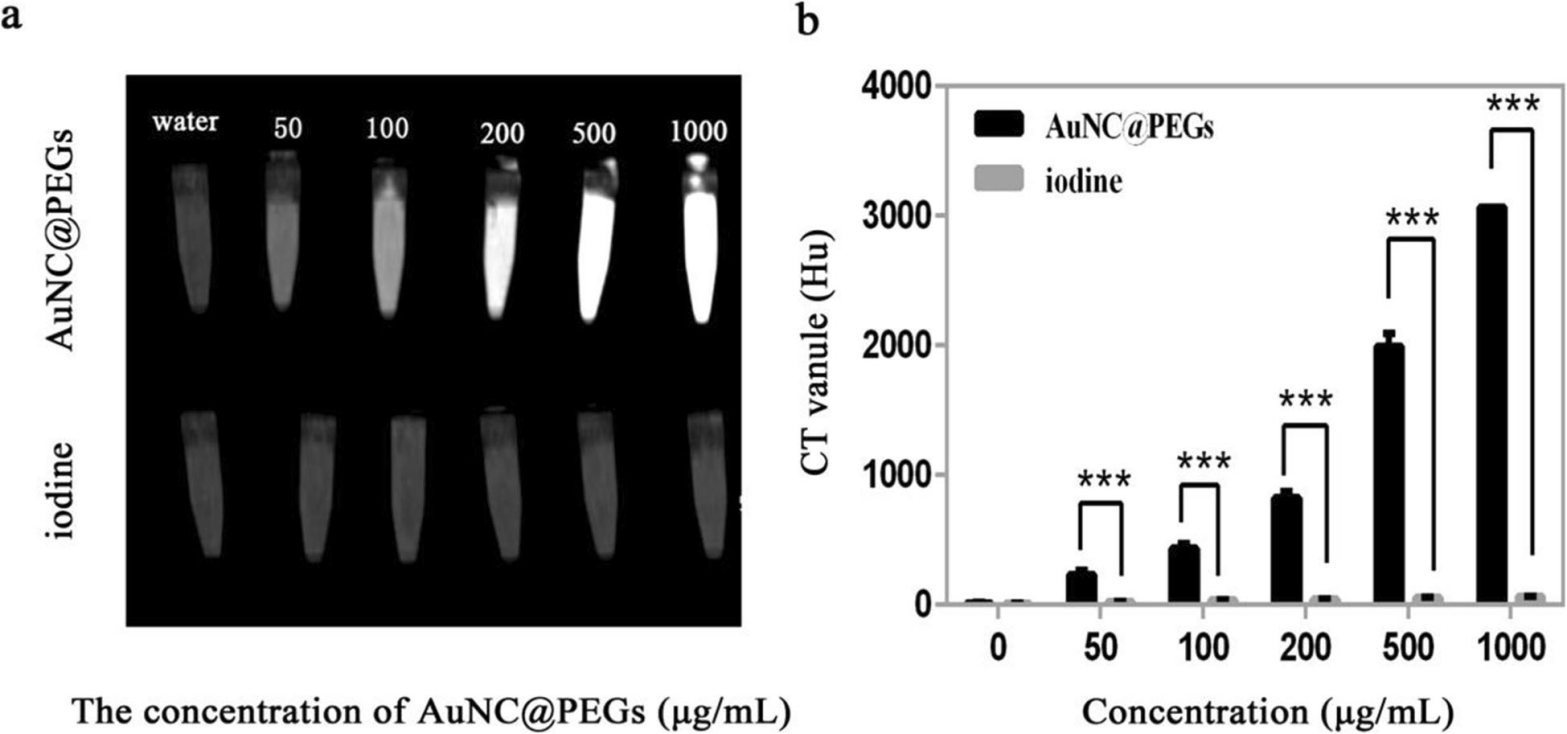
Comparison of computed tomography scan findings in vitro between AuNC@PEGs and iodine-based contrast agent. As the concentration increases, the intensity of X-ray attenuation increases (**a**). Comparison of Hu values between AuNC@PEGs and iodine-based contrast agents (**b**). ***P < 0.001

To evaluate the feasibility of the AuNC@PEGs in CT scan imaging, we compared the contrast enhancements of different molar concentrations (AuNC@PEGs) with the clinical use of a contrast agent (iodine). CT scan images were obtained, and the CT values were measured. AuNC@PEGs were compared with iodine imaging agents at similar concentrations (50, 100, 200, 500, and 1000 μg/mL). Results showed that the CT value was enhanced with the increase in concentration (Fig. 4a), and according to the analysis of the CT values of AuNC@PEGs and iodine contrast agent (Fig. 4b), the absorption coefficient of AuNC@PEGs was better than that of iodine-based contrast agents at similar concentrations, indicating that the use of AuNC@PEGs for CT scan imaging is better.

### In Vivo CT Scan Imaging

**Fig. 5:**
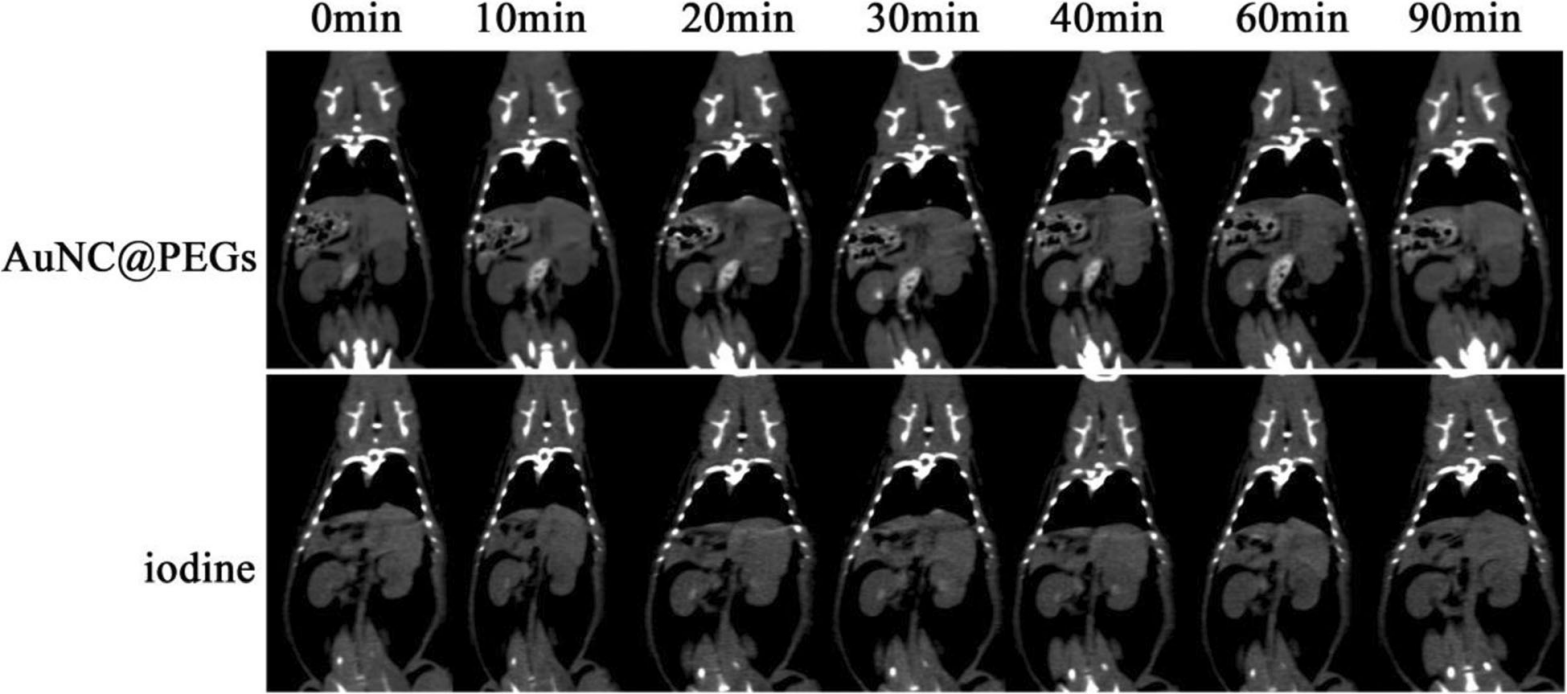
In vivo CT images of the rats at different time points after post-injection of AuNC@PEGs

Because of the high contrast ability of AuNC@PEGs, we further compared the imaging quality of AuNC@PEGs with that of iodine agents in vivo. Two hundred microliters of AuNC@PEGs (200 μg/mL) were injected via the tail vein of the rats. The time of blood pool angiography in AuNC@PEGs was evaluated via pre-injection (0 min) and post-injection continuous time point scanning (10, 20, 30, 40, 60, and 90 min). Then, the rats in the control group were injected with iodine contrast medium at an appropriate concentration. The injection dose and scanning time were similar to those of AuNC@PEGs. After the injection of a contrast agent, we simultaneously observed the contrast enhancements of the kidney (Fig. 5) and filling of the bladder (SI Fig. 1). Results showed that the kidney in the AuNC@PEGs group reached the peak at 30 min and was completely excreted at 90 min and that in the iodine-based contrast agent group reached the peak at 20 min and was completely excreted at 60 min. Then, we observed that the bladder was gradually filled with contrast agent over time. This finding showed that the time of blood pool angiography of AuNC@PEGs was better than that of the iodine-based contrast agent. The longer blood circulation time of AuNC@PEGs can provide a better diagnosis, and the AuNC@PEG has better development prospect.

### Safety of AuNC@PEGs In Vivo

**Fig. 6:**
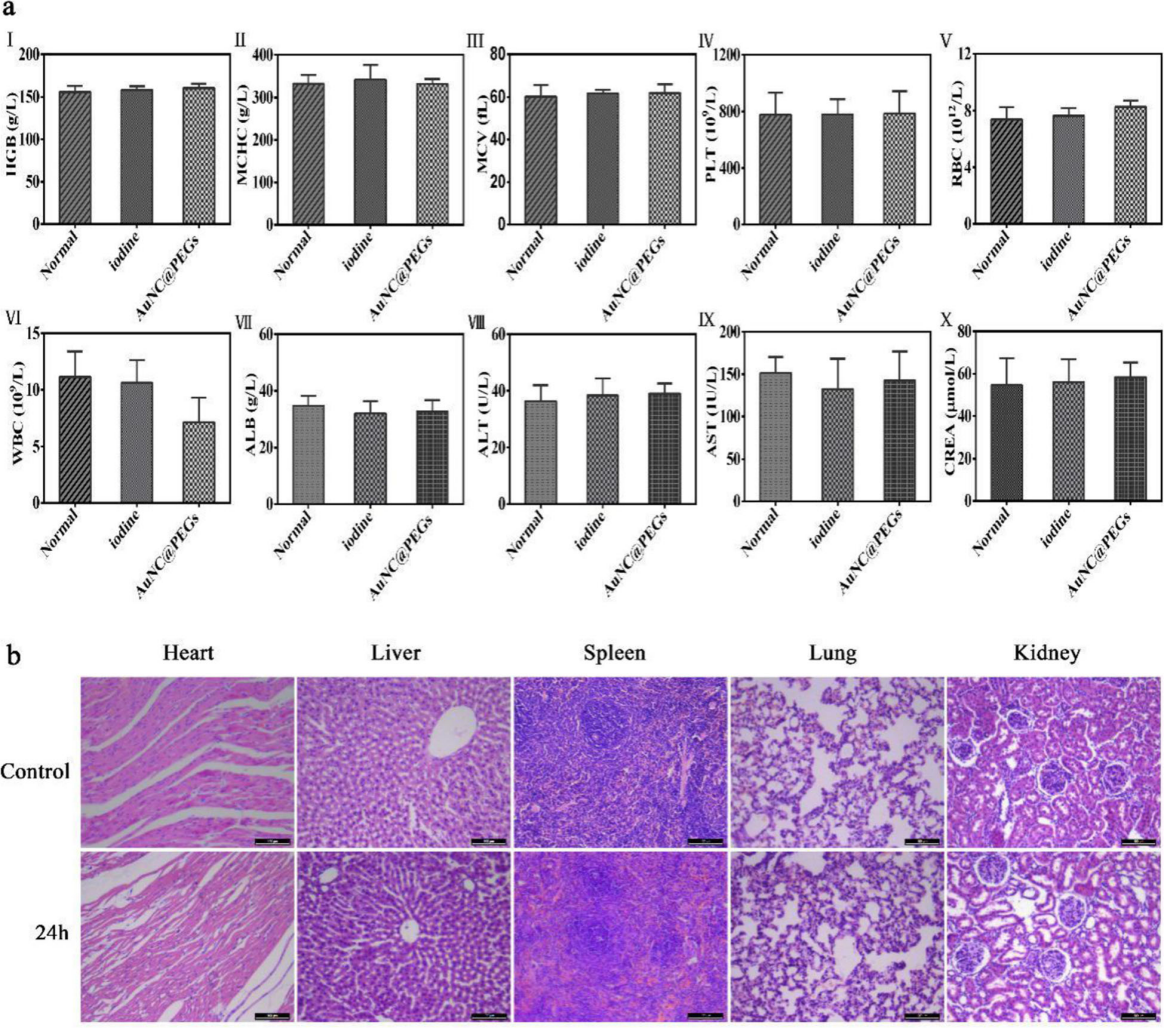
In vivo toxicity evaluation of AuNC@PEGs. Blood routine and liver and kidney function: hemoglobin level (I), mean corpuscular hemoglobin concentration (II), mean corpuscular volume (III), platelet (IV), red blood cell count (V), white blood cell count (VI), albumin concentration (VII), alanine aminotransferase level (VIII), aspartate aminotransferase level (IX), and creatinine level (X) (**a**). H&E staining was performed in the organs (the heart, liver, spleen, lung, and kidney) of normal rats and those injected with AuNC@PEGs for 24 h (**b**). The scale bars of **b** are 100 μm

As shown in Fig. 6a, the standard parameters of routine blood and liver and kidney function analyses were reflected by hemoglobin level, mean corpuscular hemoglobin concentration, mean corpuscular volume, platelet count, red blood cell count, white blood cell count, albumin concentration, alanine aminotransferase level, aspartate aminotransferase level, and creatinine level. In the statistical analysis, no significant difference was observed between the AuNC@PEG, iodine contrast agent, and control groups (*P* > 0.05). In addition, the organs (the heart, liver, spleen, lung, and kidney) of the rats were analyzed histologically, as shown in Fig. 6b, 24 h after the injection of AuNC@PEGs (200 μg/mL); sliced; and stained (H&E). Compared with the control group (not injected with nanomaterials), no obvious morphological changes and injuries were found as shown under the microscope. The abovementioned results further confirmed the safety and reliability of AuNC@PEGs in vivo.

## Conclusion

We have developed AuNC@PEG, a new type of CT scan contrast agent, with characteristics, such as small size, high contrast, long blood retention time, and low risk of toxicity. In vitro and in vivo toxicity evaluations showed that AuNC@PEGs had good biocompatibility and low risk of side effects. The imaging performance of CT scan in vitro and in vivo showed that AuNC@PEGs have a higher X-ray absorption coefficient and longer time of blood pool angiography than traditional iodine-based imaging agents. Furthermore, AuNC@PEGs are superior to iodine-based imaging agents, and the use of AuNC@PEGs is practical. All these results showed that AuNC@PEGs have a higher X-ray absorption coefficient than traditional iodine-based contrast agents and that the imaging performance of AuNC@PEGs was higher than that of traditional iodine-based contrast agents. Therefore, the synthesized functionalized AuNC@PEGs in this study have great potential for clinical application in CT scan imaging.

## Supplementary information

**Additional file 1: Figure S1.** CT imaging of bladder filling at different time points.

## Data Availability

The raw/processed data required to reproduce these findings cannot be shared at this time as the data also forms part of an ongoing research and development.

## Abbreviations

CT: Computed tomography
DCF: Dichlorofluorescein
DCFH: Dichlorofluorescin
H_2_O_2_: Hydrogen peroxide
LDH: Lactate dehydrogenase
MTT: 3-(4,5-dimethylthiazol-2-yl)-2,5-diphenyltetrazolium bromide
PBS: Phosphate-buffered saline
PEG: Polyethylene glycol
ROS: Reactive oxygen species
TEM: Transmission electron microscope
