# Supplementary material for: Use of the Highly Biocompatible Au Nanocages@PEG Nanoparticles as a New Contrast Agent for In Vivo Computed Tomography Scan Imaging
Source: Nanoscale Res Lett. 2020 Mar 4;15:53. doi: 10.1186/s11671-020-3286-2 (PMC7056796; doi:10.1186/s11671-020-3286-2)
Supplement: Supplementary file 1 — Additional file 1: Figure S1. CT imaging of bladder filling at different time points. [file 11671_2020_3286_MOESM1_ESM.docx]

**Use of the highly biocompatible Au nanocages@PEG nanoparticles as a new contrast agent for in vivo computed tomography scan imaging**

Yan Gao^1#^, Jian Kang^2#^ , Zhen Lei^1^*, Yankun Li^1^, Xifan Mei^1^* , Guannan Wang^2,3^*

^1^ The First Affiliated Hospital of Jinzhou Medical University, Jinzhou, 121001, China.

^2^ College of Pharmacy, Jinzhou Medical University, Jinzhou, 121001, China.

^3^ The Key Laboratory for Medical Tissue Engineering, College of Medical Engineering, Jining Medical University, Jining 272067, China

^#^ Both authors contributed equally to this work.

^*^ Corresponding authors:

E-mail: leizhen2004@163.com (for Pro. Lei Z.)

E-mail: meixifan1971@163.com ( for Pro. Mei X. F.)

E-mail: chemwangguannan@gmail.com ( for Pro. Wang G. N.)


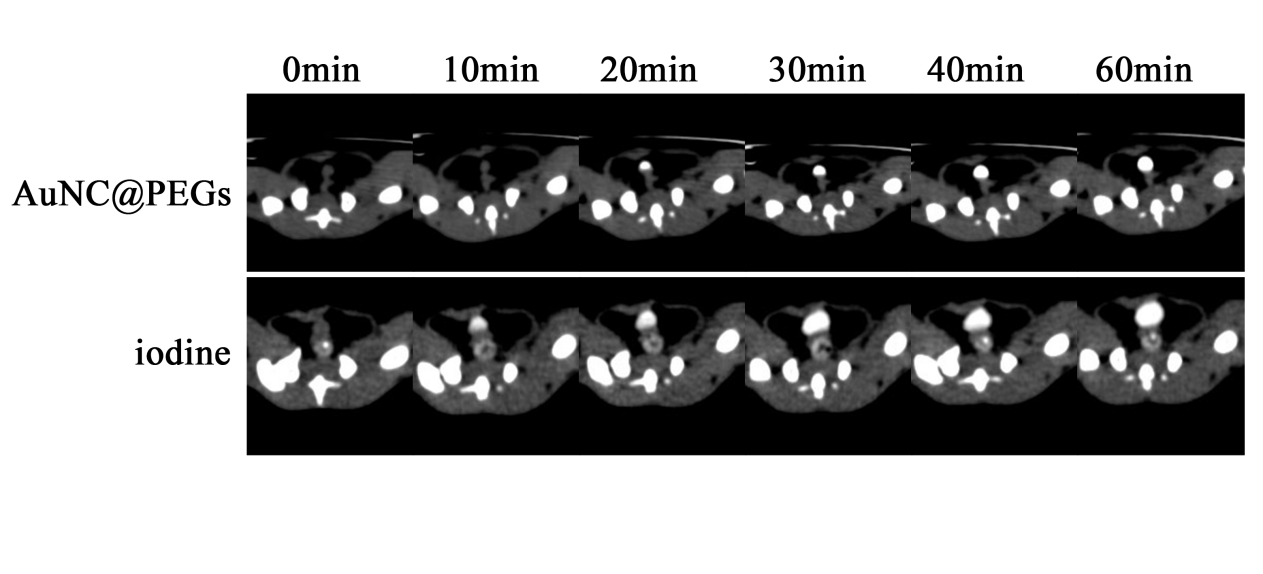


**S Figure 1.** Bladder filling at different time points (0, 10, 20, 30, 40 and 60 min).
